# C-C chemokine receptor-7 mediated endocytosis of antibody cargoes into intact cells

**DOI:** 10.3389/fphar.2013.00122

**Published:** 2013-09-24

**Authors:** Xavier Charest-Morin, Rémy Pépin, Angélique Gagné-Henley, Guillaume Morissette, Robert Lodge, François Marceau

**Affiliations:** ^1^Centre de Recherche en Rhumatologie et Immunologie, Centre Hospitalier Universitaire de QuébecQuébec, QC, Canada; ^2^Laboratory of Human Retrovirology, Institut de recherches cliniques de MontréalMontreal, QC, Canada

**Keywords:** CCR7, CCL19, melanoma, β-arrestin, Rab5, Rab7

## Abstract

The C–C chemokine receptor-7 (CCR7) is a G protein coupled receptor that has a role in leukocyte homing, but that is also expressed in aggressive tumor cells. Preclinical research supports that CCR7 is a valid target in oncology. In view of the increasing availability of therapeutic monoclonal antibodies that carry cytotoxic cargoes, we studied the feasibility of forcing intact cells to internalize known monoclonal antibodies by exploiting the cycle of endocytosis and recycling triggered by the CCR7 agonist CCL19. Firstly, an anti-CCR7 antibody (CD197; clone 150503) labeled surface recombinant CCR7 expressed in intact HEK 293a cells and the fluorescent antibody was internalized following CCL19 treatment. Secondly, a recombinant myc-tagged CCL19 construction was exploited along the anti-myc monoclonal antibody 4A6. The myc-tagged ligand was produced as a conditioned medium of transfected HEK 293a cells that contained the equivalent of 430 ng/ml of immunoreactive CCL19 (average value, ELISA determination). CCL19-myc, but not authentic CCL19, carried the fluorophore-labeled antibody 4A6 into other recipient cells that expressed recombinant CCR7 (microscopy, cytofluorometry). The immune complexes were apparent in endosomal structures, co-localized well with the small GTPase Rab5 and progressed toward Rab7-positive endosomes. A dominant negative form of Rab5 (GDP-locked) inhibited this endocytosis. Further, endosomes in CCL19-myc- or CCL19-stimulated cells were positive for β-arrestin_2_, but rarely for β-arrestin_1_. Following treatment with CCL19-myc and the 4A6 antibody, the melanoma cell line A375 that expresses endogenous CCR7 was specifically stained using a secondary peroxidase-conjugated antibody. Agonist-stimulated CCR7 can transport antibody-based cargoes, with possible therapeutic applications in oncology.

## INTRODUCTION

C–C chemokine receptor-7 (CCR7) is a G protein coupled receptor (GPCR) submitted to a cycle of endocytosis and recycling, more so if stimulated with the chemokine CCL19 than with the alternate agonist CCL21 (Otero et al., 2006). This is possibly due to the selective recruitment of β-arrestins by CCL19 (Byers et al., 2008). CCR7 is naturally expressed by naïve T cells, dendritic cells, and a subset of natural killer cells and chemotactically supports the homing of these cells to lymph node paracortex, due to the expression of CCL19 and CCL21 by lymph node stromal cells (Maghazachi, 2010; Moschovakis and Förster, 2012; Weber et al., 2013). CCR7 expression is generally upregulated during an immune response in these cells and the accumulation and retention of T cells at chronic inflammatory sites is dependent on CCR7 signaling (Moschovakis and Förster, 2012). The expression of CCR7 can evade the hemopoietic lineages in pathologies such as rheumatoid arthritis (synovial fibroblasts), scleroderma, and cancer (Müller et al., 2001; Moschovakis and Förster, 2012). First discovered in breast cancer cell lines (Müller et al., 2001), CCR7 expression has been also observed in many solid tumors and hematopoietic malignancies that have a propensity to migrate to lymph nodes. These include classical Hodgkin disease, gastric and head and neck carcinomas (Höpken et al., 2002; Mashino et al., 2002; Wang et al., 2005). Tumor cell chemotaxis mediated by CCR7 signaling is well documented and explains the metastatic homing to lymph nodes (Shields et al., 2007b; Fang and Hwang, 2009). Melanoma is a paramount example of a malignancy that acquires CCR7 expression in the metastatic state, as shown by the human A375 cell line variants: high expression of the chemokine receptor predicts the propensity for metastasis (Shields et al., 2007a).

Current preclinical research supports that CCR7 is a valid drug target in oncology (Fang and Hwang, 2009). For instance, Chemotrap-1 is a soluble macromolecule not related to CCR7, but that binds CCL21 with high affinity and prevents the metastatic extension of melanoma in animals (Lanati et al., 2010). However, the therapeutic effect of this biotechnological agent has not been demonstrated against primary tumors and established metastases.

In view of the increasing availability of therapeutic monoclonal antibodies that carry cytotoxic cargoes, we studied the feasibility of forcing intact cells to internalize known monoclonal antibodies by exploiting the cycle of endocytosis and recycling triggered by the CCR7 agonist CCL19. Firstly, an anti-CCR7 (CD197) antibody was tested in intact cells optionally stimulated with CCL19 treatment. Secondly, a recombinant myc-tagged CCL19 construction was exploited along the anti-myc monoclonal antibody 4A6. We are currently developing a platform of bifunctional myc-tagged peptide agonists of GPCRs that are suitable for coupling with both anti-myc monoclonal antibodies (such as the 4A6 clone) and their receptors. The receptor-mediated endocytosis of agonist–antibody complexes in excess of 150 kDa has been demonstrated for the bradykinin B_2_ receptor (Gera et al., 2013) and the parathyroid hormone PTH_1_R (Charest-Morin et al., unpublished data), suggesting that the approach can be generalized and exploited toward the functional cargoes. As for parathyroid hormone, but unlike bradykinin, the N-terminal sequence of CCL19 needs to be intact to preserve a good affinity for the cognate receptor (Ott et al., 2004). Thus, we have exploited a CCL19 sequence C-terminally extended with the myc epitope.

## MATERIALS AND METHODS

### CELL CULTURE, TRANSFECTION, AND ANALYSIS

A subclone of HEK 293 cells, called HEK 293a, originally obtained from Sigma-Aldrich was used in many experiments. This cell type was grown in Dulbecco’s modified Eagle’s medium (DMEM) supplemented with 10% fetal bovine serum (FBS), 1% L-glutamine, and 1% penicillin–streptomycin stock solutions (100×). HEK 293a cells were grown and transiently transfected as described (Bawolak et al., 2011) with a vector coding for CCR7 (wild type in pcDNA3, Otero et al., 2006; gift from Dr. Daniel F. Legler, University of Konstanz, Germany). Using these cells, a first CCR7 imaging strategy was based on staining intact and live HEK 293a cells previously transfected with the vector encoding for the receptor with carboxyfluorescein-conjugated monoclonal anti-human CCR7 (CD197; clone 150503, final concentration 5 μg/ml; R&D Systems, Minneapolis, MN, USA). The cell distribution of the receptor was assessed after a 30-min stimulation period with the agonist CCL19 (incubation at 37°C).

Other HEK 293a cells were used as producer cells for a secreted protein coded by vector purchased from OriGene Technologies (Rockville, MD, USA): myc-DDK-tagged prepro-CCL19, that directs the secretion of the mature human CCL19 sequence extended at its C-terminus with two epitopes in tandem, myc, and DDK (catalog number RC506523). The latter construction will be conventionally designated as CCL19-myc. Confluent producer cells (70%) were transfected with a given vector using either the ExGen (Fermentas) or TurboFect (Thermo Scientific) reagents used as directed. Conditioned medium (CM) was collected after 4 days of culture. The concentration of CCL19-myc was estimated using a commercial ELISA kit for human CCL19 (Sigma-Aldrich, catalog no. RAB0052). The CM samples were diluted 1000- to 10,000-fold for this test.

The second CCR7 visualization strategy involved recipient HEK 293a cells were grown and transiently transfected as described above with the vector coding for CCR7 and optionally co-transfected with the fusions proteins Rab5-Cherry, Rab5-GTP-locked-Cherry fluorescent protein, Rab5-GDP-locked-CherryFP, Rab7-CherryFP (given by Dr. M. J. Tremblay, Université Laval, Canada; Charest-Morin et al., 2013), or β-arrestin_1_-CherryFP (kind gift from Dr. J.-M. Beaulieu, Université Laval, Canada). Other cells were transfected with a sole vector coding for β-arrestin_2_-green fluorescent protein (GFP; gift from Dr. Michel Bouvier, Université de Montréal). Stimulations for microscopic or cytofluorometric experiments were based on the CM of the CCL19-myc construction supplemented with AlexaFluor-488-conjugated monoclonal antibodies (clone 4A6, Millipore, dilution 1:1000 corresponding to a final antibody concentration of approximately 3.3 nM in the culture medium). Cells were generally treated for 30 min with stimulants (incubation carried out at 37°C in humidified atmosphere containing 5% CO_2_), rinsed three times with phosphate buffered saline, observed in microscopy for epifluorescence, and photographed using an Olympus BX51 microscope coupled to a CoolSnap HQ digital camera (filters for GFP and AlexaFluor-488: excitation 460–500 nm, emission 510–560 nm; for Cherry fluorescent protein: excitation 525–555 nm, emission 600–660 nm). The objective lens was generally the 100× oil UPlanApo (Olympus).

Other transfected HEK 293a cells were detached using the protease-free Cell Dissociation Buffer (Invitrogen), incubated in DMEM without serum at 37°C for 30 min under agitation in the presence of a stimulant, rapidly centrifuged (30 s, 15,000 *g*) and resuspended in phosphate buffered saline. Then, the fluorescence of the cell suspensions was assessed using the BD SORP LSR II cell analyzer (BD Biosciences, Franklin Lakes, NJ, USA) for the uptake of a green fluorophore as a function of stimulation and transgene expression; results were analyzed using the BD FACS DIVA software.

### MELANOMA CELLS

The human melanoma A375, originally obtained from ATCC, was a gift from Dr. Fawzi Aoudjit (CHU de Québec, Québec, Canada). The cells were cultured in RPMI 1640 medium supplemented with 10% FBS and antibiotics at 37°C in a 5% CO_2_ humidified atmosphere. This line is originally derived from a metastatic site (Shields et al., 2007a) and is tumorigenic in immunodeficient mice (McMahon et al., 2001). To evidence the presence of CCR7, A375 cell mRNA was isolated with the Trizol reagent (Invitrogen) and was treated 30 min with DNase1 (Roche) to avoid genomic DNA contamination. cDNA were produced with 2 μg of isolate RNA and MMLV reverse transcriptase (Invitrogen). Some samples were also made without enzyme (RT-), as negative control for DNA contamination. To evaluate the presence of cell transcription of CCR7, 2 μl of cDNAs were amplified by PCR with Platinum Pfx DNA polymerase (Invitrogen, 35 cycles). Oligos used for the CCR7 amplification were: forward 5′-gcaatgggctggtcgtgttgac-3′; reverse 5′-caccttgatggccttgttgcgc-3′. Half of PCR products were revealed by agarose electrophoresis.

The agonist-stimulated uptake of the anti-myc antibody was tested as follows: A375 cells were incubated for 30 min (37°C) in the CCL19-myc or control CM supplemented with the non-labeled 4A6 monoclonal antibody (Millipore, final concentration ≈ 3.3 nM). Then, cells were fixed, permeabilized, and stained with the Tyramide Signal Amplification (TSA) Kit containing horseradish peroxidase (HRP) conjugated goat anti-mouse IgG antibodies and AlexaFluor-488-tyramide as a fluorogenic co-substrate of the reaction (Invitrogen kit T20912 used as directed). Then a microphotographic record of cell epifluorescence and transmission was obtained as outlined above.

### IMMUNOBLOTS

The agonist action of CCL19-related agonists was investigated using the expression of the transcription factor c-Fos, a distal response to the stimulation of various receptor-ligand systems (Glauser and Schlegel, 2007). Total A375 cell extracts were immunoblotted to detect c-Fos expression using the K-25 rabbit polyclonal antibodies (Santa Cruz Biotechnology; dilution 1:50,000).

### DATA ANALYSIS

Numerical values are reported as means ± SEM. Non-normally distributed groups of values were analyzed using non-parametric analysis of variance (Kruskal–Wallis test) followed by Dunn’s multiple comparison test. Normal sets of values were compared using ANOVA followed by Dunnett’s test for comparison with a common control value. In A375 cells stained with the TSA technique, the mean fluorescence intensity was determined in manually outlined cells using the Photoshop software (version 6, Adobe Systems, Mountain View, CA, USA) in multiple fields. Proportions of labeled cells or organelles in response to treatments were compared using the χ^2^ test (InStat 3.05 computer program, GraphPad Software, San Diego, CA, USA).

## RESULTS

### CCL19-INDUCED INTERNALIZATION OF CCR7-ANTI-CCR7 IN HEK 293a CELLS

**FIGURE 1:**
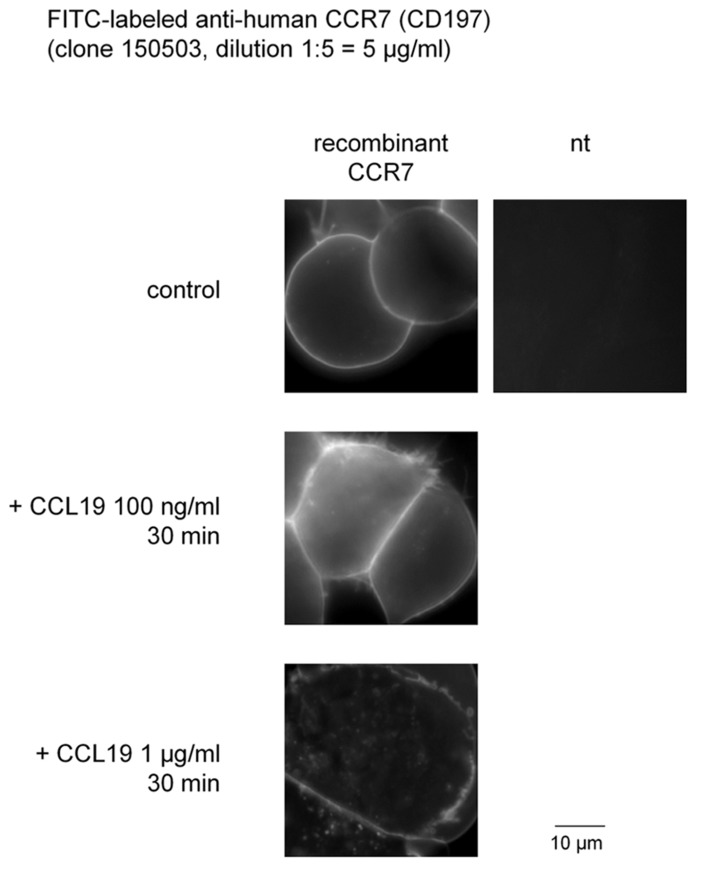
**Internalization of fluorescein isothiocyanate-conjugated monoclonal anti-human CCR7 induced by recombinant CCL19 in intact HEK 293a cells that express recombinant CCR7.** nt, non-transfected cells.

Whether a commercial anti-CD197 antibody could support imaging of CCR7 expressed in intact HEK 293a cells has been tested (**Figure 1**). In resting cells, the associated fluorescence was sharply defined at the level of the plasma membrane with no intracellular signal, as expected from the exclusion of the antibody from the cytosol. There was no signal in non-transfected cells, suggesting that the antibody reacts with extracellular domain(s) of the non-denatured CCR7. Adding CCL19 concentration-dependently determined the translocation of a fraction of the cell surface fluorescence to ill-defined cytosolic structures, consistent with agonist-induced receptor internalization (**Figure 1**).

### CHARACTERIZATION OF THE CMs

The CM of producer HEK 293a cells transfected with the CCL19-myc vector contained the equivalent of 430 ng/ml of immunoreactive CCL19 (average of three values: 150, 300, and 840 ng/ml). Three control CM of untransfected cells contained none.

### INTERNALIZATION OF myc-TAGGED CCL19-ANTI-myc IMMUNE COMPLEXES IN HEK 293a CELLS

**FIGURE 2:**
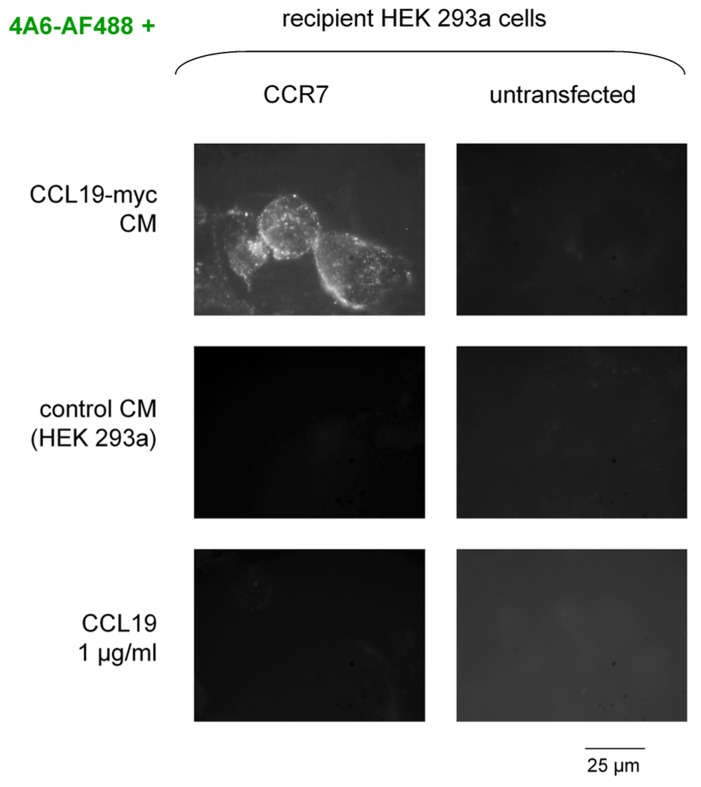
**Endocytosis of the anti-myc monoclonal antibody (clone 4A6, conjugated to AlexaFluor-488, final concentration in the culture medium 3.3 nM) as determined by co-treatment with the CCL19-myc construction in intact HEK 293a cells that optionally and transiently expressed CCR7.** A control conditioned medium (CM) or authentic CCL19 were used as control stimuli. The undiluted CM were transferred for a 30-min incubation period before rinsing and observation. Original magnification ×1000.

**FIGURE 3:**
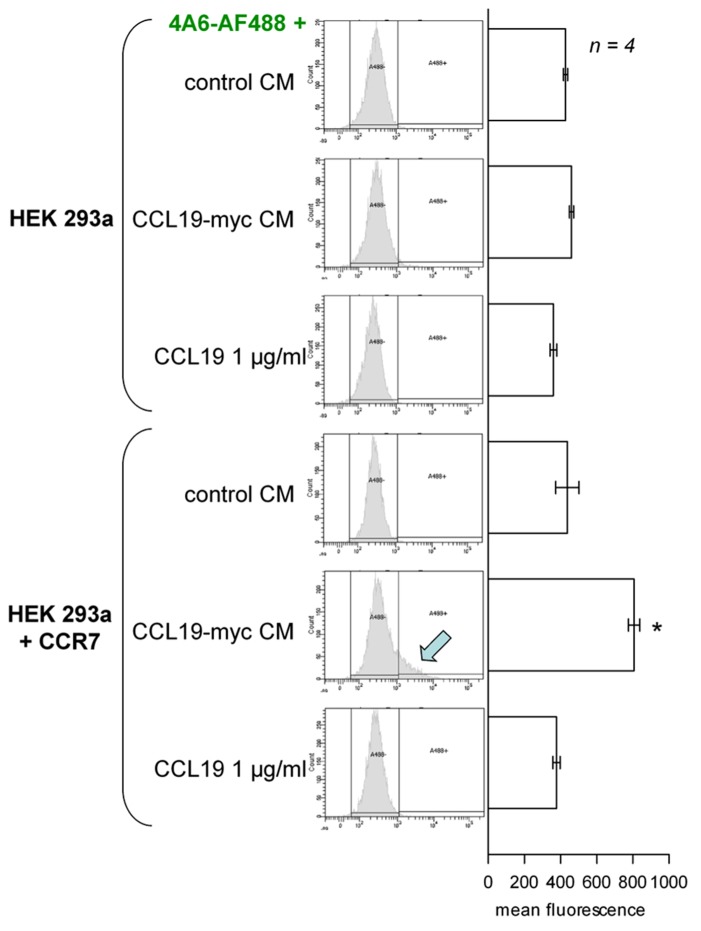
**Cytofluorometry of HEK 293a cells that optionally expressed CCR7, sequentially detached and further stimulated with the undiluted CM of other cells producing CCL19-myc, authentic CCL19, or CM of untransfected cells, as indicated (30 min incubation at 37°C).** Left: distributions based on the counting of 10,000 cells. A threshold of autofluorescence was defined using control cells with no fluorophore. It was surpassed only under one set of experimental conditions (arrow). Right: mean fluorescence of cells in replicated experiment. ANOVA indicated that the values were heterogeneous (*p* < 10^-4^). *p < 0.01 vs. top-most value (control CM, untransfected recipient cells) by Dunnett’s test.

The second scheme for the visualization of CCR7 endocytosis was based on the myc-tagged agonist co-incubated with a fluorescent anti-myc antibody in the culture medium (**Figure 2**, epifluorescence microscopy; **Figure 3**, cytofluorometry of cells sequentially detached and stimulated). Controls included cells that did not express CCR7, cells stimulated with authentic CCL19 (1 μg/ml) or with the CM of untransfected HEK 293 cells. A specific endosomal labeling of recipient cells that expressed the receptor was observed in cells stimulated with CCL19-myc (**Figure 2**), leading to a significantly higher mean cell fluorescence (**Figure 3**). These observations provide evidence that the CCL19-myc-4A6 immune complexes formed in the culture medium were recognized as agonists by the fraction of cells that expressed CCR7, and were transported into endosomes. Authentic CCL19 devoid of the myc epitope was not competent to induce the endocytosis (pinocytosis) of droplets of the culture medium containing the fluorescent antibody, providing additional evidence for the specific molecular interaction of the myc-tagged agonist with the antibody. Cells untreated with the fluorescent anti-myc antibody had mean fluorescence intensity identical to the five control conditions in **Figure 3**, suggesting that the control cells exhibited only the autofluorescence level.

**FIGURE 4:**
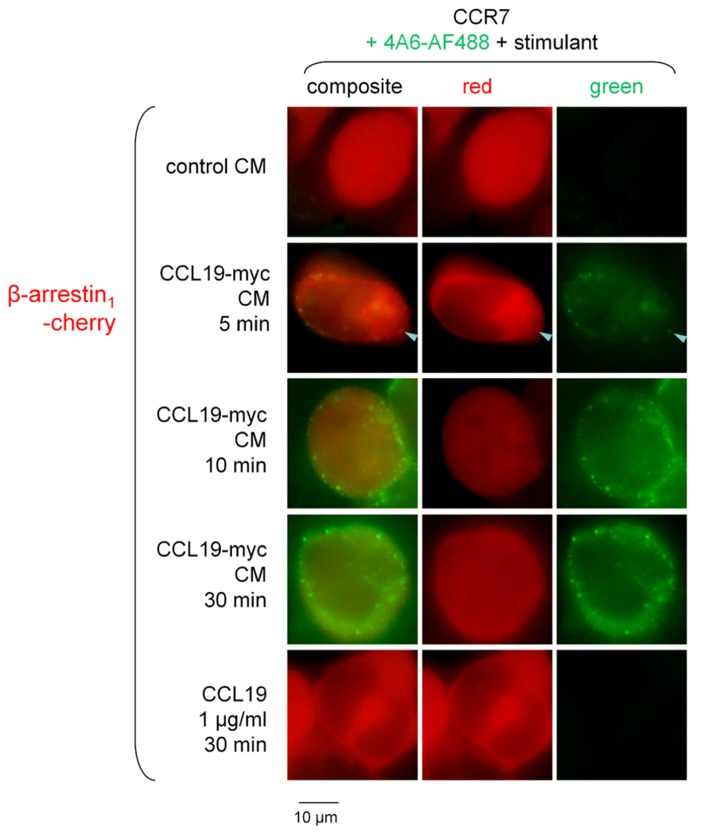
**Epifluorescence microscopy studies in cells co-expressing β-arrestin_1_-Cherry and CCR7 and stimulated as indicated (stimulant and duration).** Stimulation always included the fluorescent anti-myc monoclonal antibody 4A6 (green signal). Either CCL19-myc CM or authentic CCL19 rarely condensed β-arrestin_1_ at the level of plasma membranes of endosomes (arrowhead indicates possible co-localization with the CCL19-myc-antibody cargo). Cells that expressed well the red-emitting transgene were assumed to co-express co-transfected CCR7 and were evaluated for morphology (*n* = 16–49 eligible cells per group). The average number of red condensed structures was 0.4 ± 0.4 in cells treated with control CM and did not vary significantly in all other groups (Kruskal–Wallis test). The average number of green condensed structures in cells treated with control CM was 0 per cell and varied significantly according to treatments (*p* < 10^-4^, Kruskal–Wallis test; *p* values for Dunn’s multiple comparison test for each value vs. that of control CM reported thereafter). There were 8.3 ± 1.7 green specs per cell (*p* < 0.05), 25.8 ± 3.2 (*p* < 0.001), 33.1 ± 4.4 (*p* < 0.001), and 0 (N.S.) in cells treated with CCL19-myc CM for 5, 10, or 30 min, or with recombinant CCL19, respectively.

**FIGURE 5:**
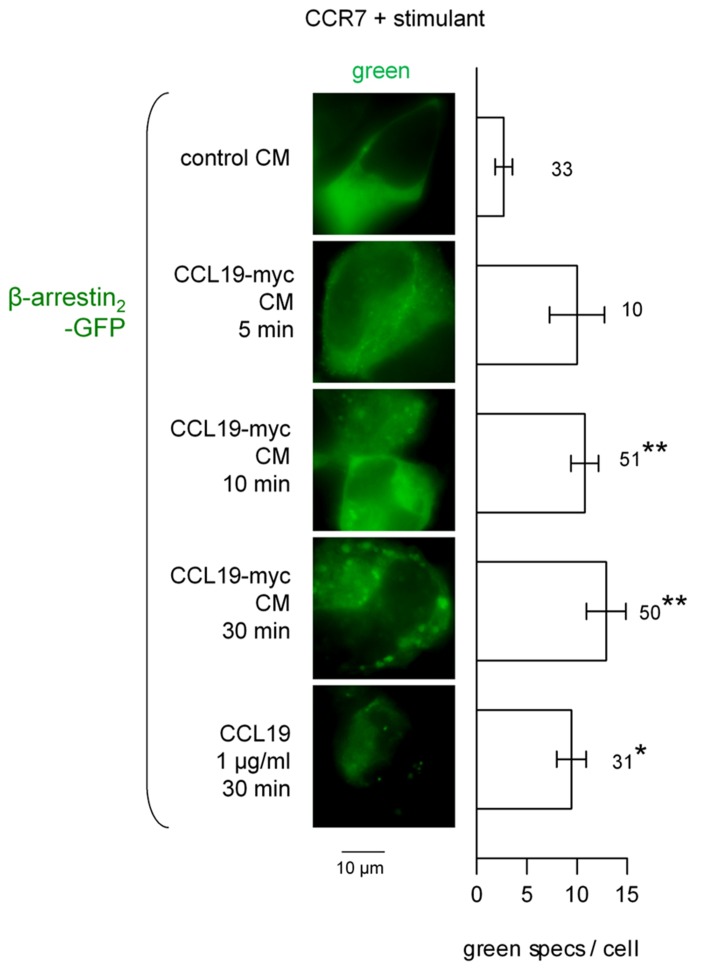
**Epifluorescence microscopy studies in cells co-expressing CCR7 and β-arrestin_2_-GFP and stimulated as indicated (stimulant and duration).** Stimulation did not include the fluorescent anti-myc antibody. Either CCL19-myc CM or authentic CCL19 frequently condensed β-arrestin_2_ at the level of plasma membrane or endosomes. Right: number of green intracellular condensed structures (“specs”) per cell ± SEM. Numbers close to bars indicate the number of evaluated HEK 293a cells. The Kruskal–Wallis test indicated that the values were heterogenous (*p* < 10^-4^). The effect of each treatment vs. the effect of control CM was evaluated using Dunn’s multiple comparison test. *p < 0.01; **p < 0.001.

The CCL19-myc/4A6 co-stimulation scheme was used to investigate β-arrestin endosomal co-localization studies (**Figure 4**). The Cherry-tagged β-arrestin_1_ is expressed as a homogeneous cytosolic protein in recipient cells that co-expressed CCR7. Neither authentic CCL19 nor CCL19-myc CM induced significant condensation of β-arrestin_1_-Cherry into endosomal structures 5–30 min after stimulation; however, the immune complexes were effectively detected at this level as the green particles (see statistics in **Figure 4** legend). The alternate construction β-arrestin_2_-GFP was co-expressed with CCR7 in other HEK 293a cells and stimulated without the 4A6 antibody (also green light emitting). It was observed that the resting smooth cytosolic distribution of this arrestin isoform was condensed into endosomal structures revealed by treatments with CCL19-myc (5–30 min) or with authentic CCL19 (**Figure 5**), producing further support for a selective affinity of activated CCR7 for one of the non-visual arrestins.

**FIGURE 6:**
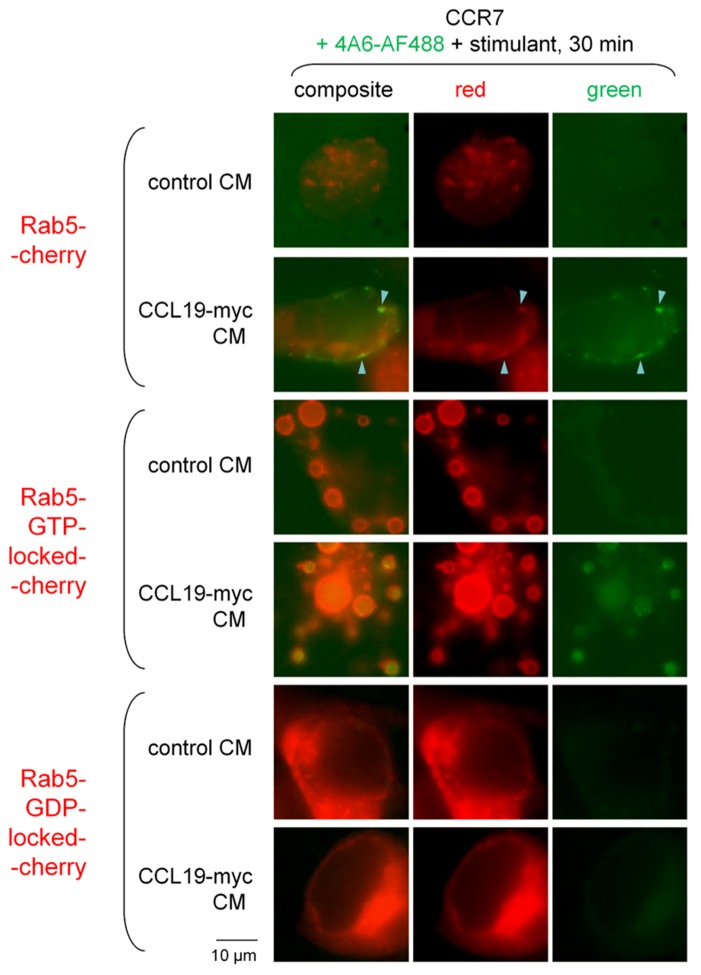
**Epifluorescence microscopy studies in cells co-expressing one of three forms of Rab5-Cherry fusion proteins and CCR7 and stimulated for 30 min with either CCL19-myc or control CM.** Co-localization between Rab5-positive corpuscles and the CCL19-myc-antibody cargo was observed (arrowheads). The dominant positive (GTP-locked) mutant of the fusion protein produced typical giant vacuoles that included the green light-emitting cargo. The dominant negative Rab5-GDP-locked-Cherry inhibited the endocytosis of the green cargo (see text for numerical analysis).

Other HEK 293a cells that expressed CCR7 were also co-transfected with one of three Rab5-Cherry constructions: the fusion protein consisting of the wild type Rab5, the GTP-locked activated one that causes the formation of giant endosomes where cargo accumulates (Stenmark et al., 1994), or the dominant negative GDP-locked Rab5 (**Figure 6**). Co-localization was observed in cells treated for 30 min with the CM of CCL19-myc in cells expressing Rab5-Cherry (arrowheads; this concerned 19.1% of green organelles in a sample of 89 from 10 cells). The GTP-locked construction induces the formation of giant vacuoles where the green fluorescence of the CCL19-myc/anti-myc-antibody complexes was extensively trapped (**Figure 6**; 88.3% of green organelles were circled by a red lining; sample of 43 from 11 cells). The GDP-locked Rab5 construction rather suppressed the endocytosis of immune complexes (average of 2.1 ± 0.9 green specs per cell in 10 cells treated with CCL19-myc CM, comparable to the value of control CM in **Figure 5**).

**FIGURE 7:**
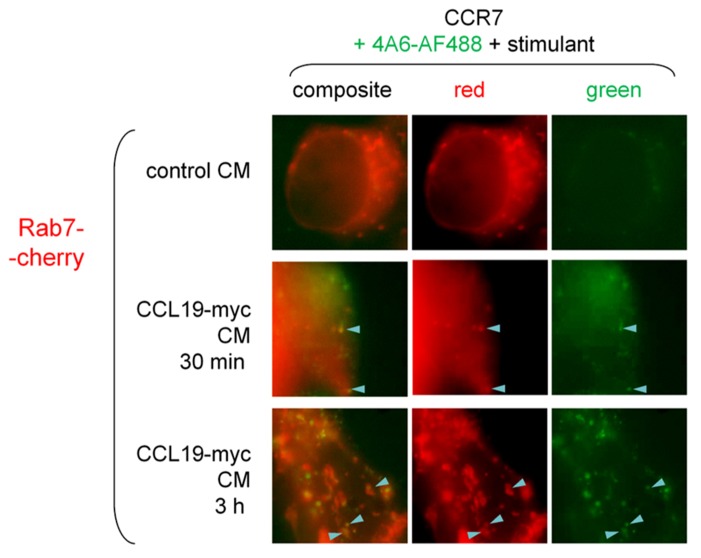
**Epifluorescence microscopy studies in cells co-expressing Rab7-Cherry and CCR7 and stimulated as indicated (stimulant and duration).** Co-localization between Rab7-positive corpuscles and the CCL19-myc-antibody cargo was not highly frequent (arrowheads), but increased in frequency as a function of incubation duration (see text for numerical analysis).

Rab5 is a marker of early endosomes, whereas Rab7 rather labels late endosomes and lysosomes. Minor co-localization of the CCL19-myc/anti-myc-antibody complexes was observed after either 30-min (5.3% of green endosomes) or 3-h treatments (12.6% of green endosomes; **Figure 7**), indicating a slow progress of the antibody cargo in the endosomal/lysosomal tract as these proportions significantly differ (*p* < 0.05, χ^2^-test; sample of 11 cells for each treatment duration containing totals of 189 and 175 green endosomes, respectively).

### CCR7 IN METASTATIC MELANOMA CELLS

**FIGURE 8:**
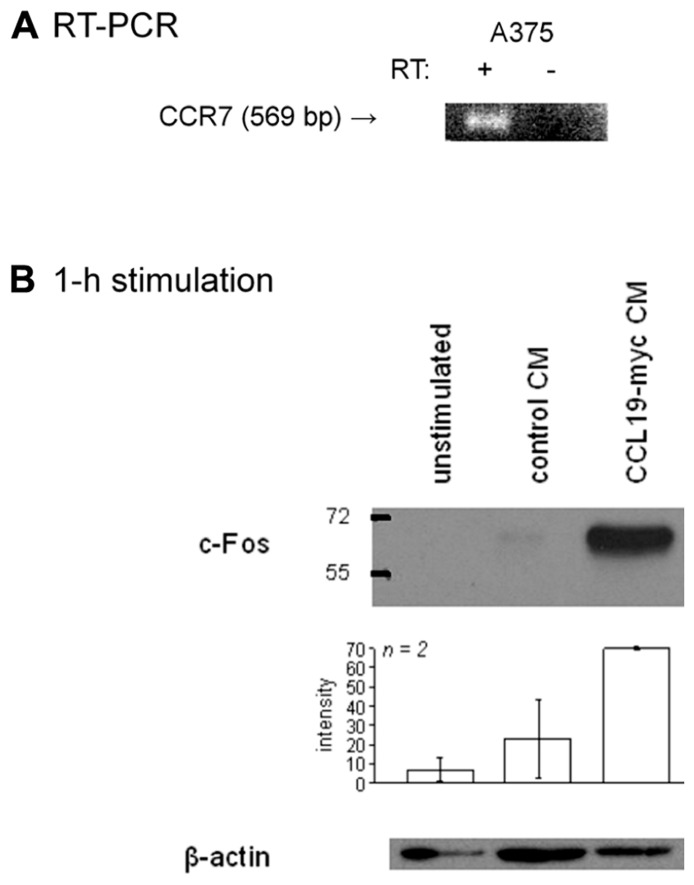
**Presence of CCR7 in A375 melanoma cells.** **(A)** RT-PCR for CCR7 in the human melanoma A375 cell line. **(B) **c-Fos induction in A375 cells stimulated as indicated for 1 h (typical immunoblot and histograms representing the densitometry of replicated experiments).

RT-PCR provided evidence for the presence of CCR7-mRNA in a human melanoma cell line, the A375 line (**Figure 8A**). The receptor is functional, as shown by c-Fos induction observed in response to 1 h of stimulation with CCL19-myc CM, but not with control CM (**Figure 8B**).

**FIGURE 9:**
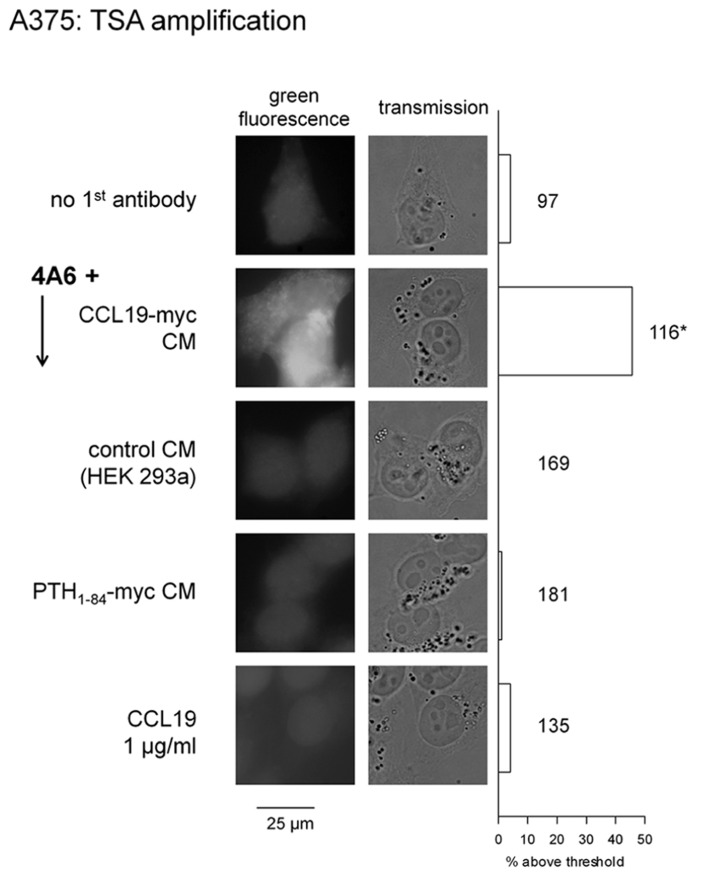
**Detection of endogenous CCR7 in A375 cells using detection of the endocytosed CCL19-myc-4A6 antibody complex using theTyramide Signal Amplification (TSA) system that enzymatically generates AlexaFluor-488 labeling.** Epifluorescence and transmission, original magnification: ×1000. Right: proportion of cells with a mean fluorescence intensity above a set threshold (Photoshop level ≥ 50/255) in large photographic records of A375 cells stained as in the left of figure (the number of evaluated cells is indicated next to each histogram). The proportions were compared with that of control cells without antibody (top-most histogram, χ^2^ test; *p < 10^-4^).

The A375 cells did not support the endosomal staining observed in HEK 293 cells that overexpress CCR7 and are co-treated with CCL19-myc + the fluorescent anti-myc antibody (as in **Figure 2**, data not shown). However, an enzymatic amplification scheme reveals the apparent specific uptake of CCL19-myc co-incubated with the non-fluorescent 4A6 monoclonal (TSA imaging: **Figure 9**). Negative controls included stimulations with the antibody combined with the control CM, authentic CCL19, or a myc-tagged parathyroid hormone, PTH_1-84_-myc (prepared as a CM in cells transfected with the OriGene vector RC519848 and containing the equivalent of ~180 ng/ml of the hormone).

## DISCUSSION

By exploiting two commercially available monoclonal antibodies, we have illustrated that the human recombinant CCR7 receptor can internalize such large proteins when stimulated with the agonist CCL19. The anti-receptor antibody clone 150503 recognized an extracellular epitope in non-denatured CCR7 expressed by intact cells (**Figure 1**) and, as such, was internalized with the stimulated receptor. Furthermore, the antibody did not inhibit the interaction of CCR7 with its agonist ligand. This experimental system is similar to that consisting of the N-terminally tagged myc-B_2_ receptor for bradykinin, that bound the anti-myc 4A6 monoclonal antibody and internalized immune complexes built around this antibody at the surface of intact cells upon stimulation with bradykinin (Bawolak et al., 2011). Data from these studies provide strong evidence that GPCR-mediated endocytosis can transport extremely large MDa cargoes, such as secondary antibodies bound to Qdot nanomaterials.

The second strategy exploited a myc-tagged agonist along with the anti-myc monoclonal antibody 4A6. It was hypothesized that the CCL19-myc construction, but not authentic CCL19, could carry anti-myc antibodies to endosomes. The myc tag is a 10-residue sequence that is widely used in recombinant protein constructs, and also in a synthetic bradykinin homolog that carried the 4A6 antibody into cells that expressed the bradykinin B_2_ receptor, albeit with low efficacy due to the low receptor affinity of the bifunctional peptide (Gera et al., 2013). The present system is more favorable as the CM of cells transfected with the CCL19-myc vector, which contains an average immunoreactive concentration (430 ng/ml), compares well with concentrations of authentic CCL19 needed to internalize CCR7 (**Figure 1**) or condense β-arrestin_2_-GFP into endosomes (**Figure 5**). Thus, the C-terminal extension of CCL19 with antigenic tags produced no significant effect on receptor affinity, as predicted from the current ligand docking model (Ott et al., 2004), and there is no apparent steric hindrance between antibody-bound CCL19-myc and the CCR7. The immune complexes, composed of a divalent antibody, may be quite large (in excess of 150 kDa) and are not taken up by activated cells by pinocytosis, as the non-tagged agonist CCL19 does not support the internalization of the 4A6 antibody added to the culture medium. Furthermore, PTH_1-84_-myc, another construction of a size comparable to CCL19-myc and also tagged at its C-terminus, failed to support endocytosis of the anti-myc antibody in A375 cells, producing evidence that an intimate trimolecular reaction (CCL19-myc + 4A6 + CCR7) at the extracellular side of the receptor is necessary for endocytosis of the antibody cargo.

C-terminal extension of CCL19 has also been illustrated under the form of a ~80 kDa dimeric CCL19-IgG Fc fusion protein that was a functional CCR7 agonist (calcium signaling, chemotaxis; Otero et al., 2006). Previous evidence, based in part on this construction and fluorescent CCR7, established that the ligand, rapidly co-localized with the receptor and transferrin in recycling endosomes, progressed slowly toward lysosomes (Otero et al., 2006). Consistent with these findings, we observed a slow transition of the 4A6 antibody initially bound to CCL19-myc to Rab7-positive organelles (late endosomes and/or lysosomes). Also, the dynamin and clathrin-dependent endocytosis of CCL19 (Otero et al., 2006) was found to be Rab5-dependent because GDP-locked Rab5 suppressed the endocytosis of the CCL19-myc-4A6 immune complexes. GDP-locked Rab5 also suppressed the endocytosis of the activated bradykinin B_2_ receptor in a recent study (Charest-Morin et al., 2013). Furthermore, receptor phosphorylation should occur prior to the internalization of CCR7 and indirect evidence of this is provided by the condensation of cytosolic β-arrestins into endosomal structures (**Figures 4** and **5**). Stimulated CCR7 may have a preference for β-arrestin_2_ over β-arrestin_1_, as the mCherry-tagged form of the latter was rarely found in endosomes where the CCL19-4A6 complexes were translocated. This asymmetry in the role of the two non-visual arrestins had been previously observed using a gene knockout approach (Byers et al., 2008) and contrasts with the activated bradykinin B_2_ receptor, equally capable of associating with both forms (also used as fluorescent protein conjugates; Gera et al., 2012).

The metastatic melanoma A375 cell line possesses a functional endogenous population of CCR7 (**Figure 8**; Shields et al., 2007a) that also supported the uptake of a small amount of anti-myc antibody complexed to CCL19-myc, relative to cells that expressed recombinant CCR7 at high density. At the risk of temporarily enhancing migration, survival and invasion (Fang and Hwang, 2009), but perhaps with the benefit of sensitizing cells to anti-mitotic agents, CCR7 stimulation with a tagged agonist in oncology may allow the β-arrestin-dependent endocytosis of a cytotoxic cargo, such as an antibody conjugated to maytansinoids or others high potency cytotoxic drugs. This could kill tumor cells with some selectivity if the cytotoxic cargo is allowed to enter the endocytic pathway even in very small quantities (Teicher and Doroshow, 2012). An alternative application could be the killing of CCR7-positive dendritic cells, which form long lasting reservoirs for HIV that preclude viral eradication (Zhang and Perelson, 2013), and of infected CD4^+^ T cells, in which CCR7 activation may contribute to the nuclear integration of the HIV genome (Cameron et al., 2010).

In addition to the endocytosis of anti-CCR7 antibody triggered by CCL19, the present studies indicate the feasibility of the specific cellular uptake of an immune complex consisting of a tagged agonist of CCR7 and of the anti-myc antibody. The latter approach is essentially modular, as the same anti-myc antibody can be transported in cell expressing various types of receptors by varying the identity of the myc-tagged agonist (Gera et al., 2013; Charest-Morin et al., unpublished data). One can imagine a form of personalized treatment where the tagged agonist is selected according to the repertoire of internalized receptors, whether or not GPCRs, expressed by a given malignancy.

## Conflict of Interest Statement

The authors declare that the research was conducted in the absence of any commercial or financial relationships that could be construed as a potential conflict of interest.
